# Protracted primary cytomegalovirus infection presenting as ileoanal pouchitis in a non-immunosuppressed patient: a case report

**DOI:** 10.1186/1752-1947-8-163

**Published:** 2014-05-26

**Authors:** Christian Rupp, Esther Herpel, Paul Schnitzler, Anna Zawierucha, Philipp Zwickel, Lukas Klute, Martina Kadmon, Wolfgang Stremmel, Annika Gauss

**Affiliations:** 1 Department of Gastroenterology and Hepatology, University Hospital Heidelberg, INF 410, 69120 Heidelberg, Germany; 2 Department of Pathology, University Hospital Heidelberg, INF 224, 69120 Heidelberg, Germany; 3 Department of Infectious Diseases, Virology, University Hospital Heidelberg, INF 324, 69120 Heidelberg, Germany; 4 Department of Surgery, University Hospital Heidelberg, INF 110, 69120 Heidelberg, Germany

**Keywords:** Cytomegalovirus, Pouchitis, Primary infection, Ulcerative colitis

## Abstract

**Introduction:**

Pouchitis often occurs after proctocolectomy and ileal pouch-anal anastomosis for ulcerative colitis. It is usually deemed idiopathic and commonly responds to antibacterial therapy. To date, only a few cases of cytomegalovirus pouchitis have been documented, and only a single report describes pouchitis in a case of assumed primary cytomegalovirus infection.

**Case presentation:**

A 26-year-old Caucasian woman underwent proctocolectomy and ileal pouch-anal anastomosis for refractory ulcerative colitis and adenocarcinoma. After 28 months she developed bloody diarrhoea, abdominal pain, fever, nausea and general malaise suggesting severe pouchitis. Antibiotic treatment reduced humoral inflammation, but failed to resolve her fever. A pouchoscopy revealed distinct pouchitis, and cytomegalovirus infection was diagnosed from pouch biopsies by polymerase chain reaction as well as conventional histology and immunohistochemistry. The infection was confirmed in her blood by polymerase chain reaction and pp65 antigen test, and was clearly defined as the ‘primary’ infection by serial serological tests. Intravenous treatment with ganciclovir (10mg/kg body weight/day) led to resolution of symptoms and negative cytomegalovirus deoxyribonucleic acid and pp65 within a few days. When symptoms and laboratory evidence of cytomegalovirus infection recurred a few days after completing 20 days of therapy with ganciclovir and valganciclovir, a second course of ganciclovir treatment was initiated.

**Conclusions:**

Cytomegalovirus infection of the ileoanal pouch is an important differential diagnosis of pouchitis even in non-immunosuppressed patients and can be treated with ganciclovir.

## Introduction

Idiopathic pouchitis is a common problem after ileal pouch-anal anastomosis (IPAA) for ulcerative colitis (UC). It can usually be managed with antibiotics [1]. More rarely, specific causes of pouchitis can be identified, for example *Clostridium difficile* or cytomegalovirus (CMV) infection [1-6]. CMV has infected between 40 and 100% of the adult population [7-9]. This facultative pathogen causes clinical illness in a small percentage of the infected, with the greatest risk during the intrauterine period or in immunocompromised patients [10].

CMV can aggravate pre-existing inflammatory bowel disease (IBD), especially in immunosuppressed patients, whereas CMV colitis has only rarely been described in immunocompetent patients without IBD [6,11-13]. There are few reports on CMV infection after restorative proctocolectomy in patients with UC. A single case of a patient with putative ‘primary’ CMV infection of the pouch was published in 1998, and the patient was successfully treated with ganciclovir [4]. In that case report, the definition of primary CMV infection versus reactivation was derived from acute symptoms, pouch biopsies with positive immunohistochemistry and the presence of CMV immunoglobulin (Ig) M and G antibodies in the blood. Since no data on antibody avidity or serological analyses from samples taken prior to the onset of symptoms were presented, a reliable differentiation from CMV reactivation is not possible. Two other case reports describe chronic refractory pouchitis due to CMV infection [14]. Very recently, McCurdy *et al.* published a retrospective case series of seven patients with CMV infection of the ileoanal pouch, of whom five were immunocompromised, most of them being liver transplant patients for primary sclerosing cholangitis. In that paper, it was not stated whether any of the patients suffered from primary CMV infection [6]. The only systematic analysis on the prevalence of CMV in the ileoanal pouch detected CMV genes and proteins in 41.6% of patients with pouchitis after proctocolectomy for UC, but only in 11.2% of otherwise comparable patients with normal pouch findings [5]. Recently, Tribonias *et al.* raised the question of whether CMV in pouch mucosa might be the ‘real enemy or the innocent bystander’ [3]. The answer may be influenced by case-specific factors and even then difficult to determine conclusively.

Here, we report a rare case of primary CMV infection with pouchitis as the prevailing disease manifestation in a patient after proctocolectomy and IPAA for UC.

## Case presentation

In 2011, a 26-year-old Caucasian woman with concomitant coeliac disease underwent proctocolectomy and IPAA for refractory UC and adenocarcinoma of the colon. She did not undergo chemotherapy. Three months later, her ileostomy was closed. After 1 year, she suffered from acute pouchitis, which was successfully treated with ciprofloxacin and metronidazole.

She was admitted to our hospital 2.5 years after her proctocolectomy, with severe diarrhoea, abdominal cramps, discharge of blood, nausea, emesis, generalised malaise and chills with fever up to 38.5°C. Prior to admission, she was not taking any medication. The physical examination showed tachycardia (104 beats per minute) and tenderness of her lower abdomen. Laboratory data revealed high C-reactive protein (CRP 117mg/L: <5mg/L) and lactate dehydrogenase (424U/L: <248U/L) and slight leucocytosis (11.7/nL: <10.0/nL) with atypical lymphocytosis (Table 1). Urine analysis, electrocardiography, abdominal ultrasound and X-ray of her chest were unremarkable. The result of the stool analysis for pathogenic bacteria and viruses was negative, as were bacterial blood cultures. As idiopathic pouchitis was suspected, ciprofloxacin and metronidazole were administered. Antibiotic treatment resulted in a decrease of CRP, but no normalisation.

**Table 1:** Laboratory markers and cytomegalovirus load from blood and pouch biopsies at six time points in the course of the patient’s disease

**Time point**	**CRP (mg/L)**	**Blood leukocytes (/nL)**	**LDH (U/L)**	**CMV DNA in serum (copies/mL)**	**CMV IgM (positive or negative); CMV IgG (titre)**	**CMV DNA in pouch biopsy (copies/mL), at different sites**
Before first course of ganciclovir (day 2 of first admission)	117	11.7	424	10,300	Positive; 1:8200	7.3×10^7^
During first out-patient visit, after 20 days of therapy with ganciclovir and valganciclovir	18	9.4	222	Negative	Positive; 1:13,000	<1000 (outside ulcer)
During second hospital stay	13.7	7.7	214	2500	n. d.	83,700 (from ulcer); 1050 (outside ulcer)
2 weeks after discharge from the second hospital stay	8	8.4	249	Negative	Positive; 1:26,000	n. d.
7 weeks after discharge from the second hospital stay	2.8	10.9	187	Negative	n. d.	8860; 13,500; 34,900 (no ulcer)
10 weeks after discharge from second hospital stay	2.4	8.3	183	Negative	n. d.	3×negative (no ulcer)

*Abbreviations:* CMV, cytomegalovirus; DNA, deoxyribonucleic acid; IgM, immunoglobulin M; IgG, immunoglobulin G; CRP, C-reactive protein; Ig, Immunoglobulin; LDH, lactate dehydrogenase; n. d., not done. Normal ranges of laboratory parameters were: CRP <5mg/L; leukocytes 4–10/nL; LDH <248U/L.

**Figure 1:**
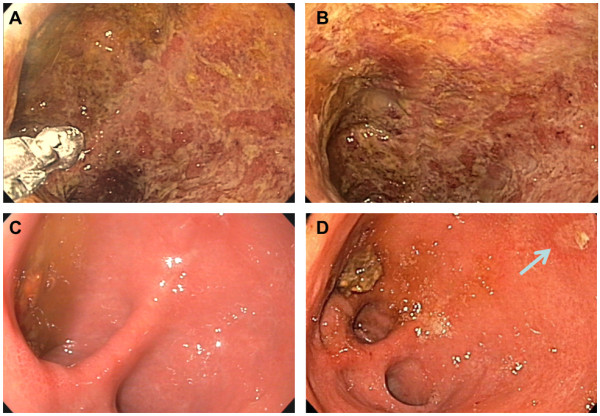
**Pouchoscopy findings before and after ganciclovir therapy. A** and **B** show endoscopic photographs of the pouch one day after admission to our hospital before treatment with ganciclovir; note the size of the biopsy tweezers in the lower left corner of **A**. **C** shows a comparable view into the pouch 25 days after the end of the first hospital stay and after treatment with ganciclovir and valganciclovir. At that time point, the mucosa was smooth and shiny within the whole pouch except for a single small ulceration (about 3×7mm) in the pouch corpus; **D** shows the view into the pouch on day 6 of the second admission to our hospital. The ulceration, marked with an arrow, is clearly healing.

**Figure 2:**
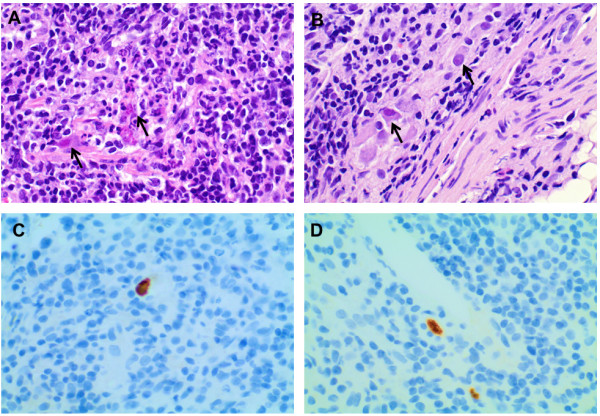
**Microscopic findings in cytomegalovirus pouchitis. A** and **B** show microphotographs of pouch biopsies taken before the first course of treatment with ganciclovir (hematoxylin and eosin stain, ×40). Note the enlarged nuclei of some cells and the virus-induced eosinophilic intranuclear and cytoplasmatic inclusions (arrows). **C** and **D**: immunohistochemistry revealing positive staining for cytomegalovirus (×40).

A pouchoscopy on day 2 revealed pronounced pouchitis with loss of vascularity, erythema, erosions, friability of the mucosa and profuse fibrin exudates (Figures 1A and 1B). Microscopic analysis showed severe erosive inflammation with numerous granulocytes (Figure 2). CMV polymerase chain reaction (PCR) from pouch biopsy (available on day 5) yielded a viral burden of 7.3×10^7^ copies/mL. A hematoxylin and eosin stain revealed CMV inclusion bodies, while immunohistochemistry showed strong nuclear staining for CMV antigen (Figure 2). CMV deoxyribonucleic acid (DNA) and CMV pp65 antigen in her blood were 10,300 copies/mL and 4/500,000 cells, respectively (Table 1).

On day 5, intravenous (IV) antiviral therapy with ganciclovir (10mg/kg/day) was initiated. CMV pp65 in her blood was negative after five infusions of ganciclovir and remained so until she was discharged. Her symptoms resolved gradually, and CRP decreased. On day 10 of IV antiviral treatment, she was discharged in good clinical condition. Antibiotic treatment was stopped. Oral antiviral treatment with valganciclovir (2×900mg/day) was prescribed for another 10 days, followed by prophylactic treatment (2×450mg/day) until the scheduled follow-up.

To differentiate between primary CMV infection and reactivation, archived blood samples were analysed for anti-CMV antibodies. CMV PCR and CMV immunohistochemistry were performed on previously acquired formalin-fixed pouch specimens and the proctocolectomy specimen. Serum CMV IgM and IgG antibodies were detected for the first time during the patient’s hospital stay; they were found to be negative in retrospective analyses of archived serum specimens taken in 2009, 2010 and 2011. Avidity of CMV IgG was low, consistent with primary CMV infection. CMV also tested negative in the archived biopsies. Thus primary CMV infection could be confirmed for certain.

She reported, 25 days after she was discharged from our hospital, a sense of well-being, aside from a persistent dry cough and increasing dyspnoea which had started shortly after initiation of ganciclovir treatment. Examination of her lungs yielded expiratory wheezing on both sides. A pouchoscopy revealed normal findings except for a single small ulcer in the pouch corpus (Figure 1C). CMV DNA in the pouch tissue (outside the ulcer) was <1000 copies/mL, whereas CMV pp65 and CMV DNA in her blood tested negative (Table 1). Due to suspected pulmonary side effects of valganciclovir, treatment was paused. The patient reported decreased cough and dyspnoea 2 days later; because there was a primary CMV infection in a non-immunosuppressed patient, we decided not to resume therapy.

Ten days later, she reported increased frequency of bowel movements and abdominal pain. CMV DNA in her blood tested positive again, with 2500 copies/mL. We decided to readmit her to the ward for another 16 day course of IV ganciclovir treatment and concomitant treatment of her cough, which increased again after resuming antiviral therapy. There was no evidence of lung embolism, tuberculosis, human immunodeficiency virus infection or infection with *Mycoplasma*, *Pneumocystis*, *Chlamydia*, *Legionella* or *Aspergillus*. CMV PCR was negative in her sputum. The results of an X-ray of her chest and pulmonary function tests were unremarkable. A pouchoscopy on day 6 of readmission showed a very small healing ulcer in otherwise macroscopically and microscopically normal mucosa (Figure 1D). A biopsy taken from the ulcer revealed positive CMV DNA with 83,700 copies/mL; another biopsy from macroscopically normal mucosa contained 1050 copies/mL. CMV PCR in her blood was already negative 3 days after restart of treatment. She was discharged in good clinical condition with ongoing valganciclovir treatment (2×900mg/day) and symptomatic therapy with salbutamol and codeine.

At the out-patient follow-up, she had completed another 14-day course of valganciclovir (2×900mg/day) and started prophylactic therapy (2×450mg/day) 3 days prior. She reported being well except for a cough which was treated with the above-indicated symptomatic medication. CMV PCR and CMV pp65 in her blood remained negative. Markers of humoral inflammation were nearly normal (Table 1). Prophylactic valganciclovir therapy was continued. The next pouchoscopy 5 weeks later, with the patient still undergoing valganciclovir prophylaxis, showed no ulcerations. CMV DNA was detected at three different sites of the pouch (8860 copies/mL, 13,500 copies/mL and 34,900 copies/mL), but no inclusion bodies were identified, and immunohistochemistry was negative. CMV pp65 and CMV PCR in her blood were again negative. We decided to discontinue valganciclovir prophylaxis because there were no relevant symptoms of pouchitis at that time and, in addition to her cough, she developed pruritus, and we found elevated alanine aminotransferase (ALT) (216U/L: <35U/L) in her blood. In response to an increasing number of bowel movements, we initiated oral rifaximin therapy (2×550mg/day for 2 weeks) with the intent to strengthen the mucosal barrier of the pouch and thus to support her immune response against CMV even without specific antiviral treatment. Her liver enzymes normalised quickly, and her cough improved, but did not resolve completely. Another pouchoscopy 3 weeks after the last one revealed macroscopically normal mucosa, negative CMV genome detection at three different sites of the pouch, and negative CMV immunohistochemistry, as well as no CMV inclusion bodies. During the visit at our outpatient clinic, she complained about symptoms of pouchitis no longer.

## Discussion

We present a case of primary CMV infection of the ileoanal pouch in a non-immunosuppressed patient after restorative proctocolectomy for UC. To the best of our knowledge, only one other case of primary CMV infection manifesting as pouchitis has been published [4], although primary infection was not clearly established there.

The route of transmission in our patient could not be traced. The symptoms of our patient were suggestive of a flare of idiopathic pouchitis, especially given that she had previously experienced an episode of pouchitis with successful antibiotic treatment. However, during the previous episode, she had not suffered from fever, abdominal cramps, or bloody stools. This raised the suspicion of an additional specific pathogen, and prompted us to test for CMV in pouch biopsies. Macroscopic alterations of the pouch mucosa are not specific for CMV infection. Endoscopic findings in CMV infection of the gastrointestinal tract comprise mucosal oedema, erythema, friability, granularity, and ulceration, and are not discernible from findings due to other causes such as UC [14-16]. Neutrophil inflammatory exudate in the pouch mucosa, as seen in our patient, has previously been described as a histopathological finding of CMV pouchitis [14]. As CMV inclusion bodies are not always visible, CMV pouchitis can sometimes only be excluded when suspected by the treating doctor, who has to request CMV immunohistochemistry or CMV PCR.

Intestinal CMV infection is rare in immunocompetent patients [17-21]. The systemic primary CMV infection manifesting as pouchitis in our patient raised the suspicion that there may have been some sort of local immunosuppression in the pouch. The finding that antibacterial therapy was already partly successful before antiviral therapy was first initiated and that rifaximin treatment apparently helped CMV clearance later on, indicate that bacterial overgrowth disturbs mucosal integrity of the pouch and thus increases the probability of viral infection and persistence. It is likely that in our patient, CMV infection complicated pre-existing idiopathic pouchitis, even though it was no reactivation, but a primary infection.

We suggest that in our patient, CMV was not just an ‘innocent bystander’, but a potentially relevant ‘enemy’. The initial decision to begin antiviral treatment was based on the general malaise of the patient, the relatively high viral burden in the biopsies, and positive CMV DNA and pp65 in her blood. We chose to treat the infection for 10 days with IV ganciclovir and switched to a therapeutic dose of oral valganciclovir for another 10 days. This approach is in line with the treatment recommended in national and international UC guidelines [22,23]. Usually, a 2- to 3-week course of ganciclovir alone or ganciclovir and subsequent valganciclovir is recommended. There are no consistent recommendations on the need and duration of post-therapy intake of valganciclovir, especially not in cases of primary CMV infection in immunocompetent patients. Of interest, in our patient, the ileoanal pouch was clearly the main reservoir of CMV. Obviously, CMV infection can persist much longer in the intestinal mucosa than suggested from blood results. Because of this, the treatment duration of 2 to 3 weeks may be too short and, as suggested by McCurdy *et al.*[6], not only blood tests, but also a repeat endoscopic examination should be considered to confirm CMV eradication and to assess mucosal healing. In our patient, we feel that the initial treatment duration was too short. Of course, the situation in our patient was peculiar, as she developed relevant side effects of the antiviral therapy. But the time span between the first diagnosis of CMV pouchitis and the last pouchoscopy – in which CMV could not be detected in the pouch mucosa – was 140 days, despite the patient receiving antiviral treatment or prophylaxis during much of this period. In retrospect, it might have been helpful if we had adhered to antibiotic treatment with ciprofloxacin and metronidazole in addition to antiviral treatment following discharge from the first hospital stay, e. g. for 4 weeks.

Also, our case reveals once more the importance of the selection of the site of the mucosa from which to take a specimen for CMV DNA analysis because samples taken from ulcers contain significantly more CMV DNA than those taken from intact mucosa.

## Conclusions

In conclusion, CMV should be considered a cause of pouchitis, given the availability of an efficient antiviral treatment, especially in patients suffering from fever. CMV infection of the pouch might be a complication of pre-existing idiopathic pouchitis, so that additional antibacterial treatment seems to be helpful. A prospective study to systematically analyse the role of CMV in pouchitis is worthwhile.

## Consent

Written informed consent was obtained from the patient for publication of this case report and accompanying images. A copy of the written consent is available for review by the Editor-in-Chief of this journal.

## Abbreviations

CMV: Cytomegalovirus; CRP: C-reactive protein; IBD: Inflammatory bowel disease; Ig: Immunoglobulin; IPAA: Ileal pouch-anal anastomosis; IV: Intravenous; PCR: Polymerase chain reaction; UC: Ulcerative colitis.

## Competing interests

The authors declare that they have no competing interests.

## Authors’ contributions

CR and LK acquired patient data. CR was involved in drafting the manuscript. AG acquired patient data and drafted the manuscript. EH was responsible for all histopathological analyses and provided the images. PS performed virological analyses. AZ, PZ, MK and WS were also actively involved in preparing the manuscript and critical appraisal. All authors read and approved the final manuscript.
